# Factors associated with influenza vaccination compliance at a tertiary care teaching hospital in Argentina

**DOI:** 10.1186/2047-2994-4-S1-P92

**Published:** 2015-06-16

**Authors:** RE Quirós, A Novau, L Davide, A Parisotto

**Affiliations:** 1 Prevention and Infection Control Department, Hospital Universitario Austral, Pilar, Argentina; 2 Pharmacy Department, Hospital Universitario Austral, Pilar, Argentina; 3 Nursing Department, Hospital Universitario Austral, Pilar, Argentina

## Introduction

After pandemic influenza A H1N1 in 2009, an increase in influenza vaccination compliance among healthcare personnel was evident in all care settings. However, these levels could not be sustained over time. Although different variables are associated with the level of compliance to influenza vaccination, these have not been evaluated at institutional level in our country. Their identification could allow adjustments in influenza vaccination campaigns in order to increase its compliance.

## Objectives

To identify factors associated with influenza vaccination compliance during a multiyear period of intervention.

## Methods

A retrospective observational study, at a 142-bed tertiary care teaching hospital, was conducted to evaluate those factors associated with influenza vaccination compliance. All staff who worked for at least four years between March 2010 and June 2014 was included in the study. As influenza vaccination is mandatory for staff in our institution, Department for Infection Prevention and Control implemented annual campaigns to increase the compliance with this recommendation based on brochures, reminders and vaccination in the workplace.

## Results

There were 6,612 opportunities of influenza vaccination with an overall compliance of 75.8% (95% CI 74.8% – 76.9%). Significant differences in compliance were observed between ancillary staff (94.0%), nurses (91.7%), and physicians (50.9%). Females have better compliance (83.2%) than males (62.8%), and employees (87.5%) than contract personnel (46.8%). Influenza vaccination decrease significantly from age 19 - 29 years (90.7%) to age ≥ 60 years (53.5%). Except sex, the remaining variables persisted in the controlled multivariate model for compliance. The overall rate of compliance decreased from 76.9% in the first year of vaccination to 71.4% in the final year, reaching a peak of 82.2% in the second year.

## Conclusion

Despite an acceptable level of compliance immediately after the pandemic, the study allowed to demonstrate a significant reduction in vaccination rates in recent years. Since, medical staff, and contract personnel represent the groups with less influenza vaccination compliance, target strategies should be developed to improve the compliance of future influenza vaccination campaigns.

## Disclosure of interest

None declared.

